# Supplementary material for: Random walk informed heterogeneity detection reveals how the lymph node conduit network influences T cells collective exploration behavior
Source: PLoS Comput Biol. 2023 May 24;19(5):e1011168. doi: 10.1371/journal.pcbi.1011168 (PMC10243635; doi:10.1371/journal.pcbi.1011168)
Supplement: S1 Text — (PDF) [file pcbi.1011168.s001.pdf]

S1 Text Detailed spectral decomposition of the transition matrix

T is non symmetric so its spectral decomposition is not guaranteed. Let $T_s = D^{1/2} T D^{-1/2}$ the symmetrized transition matrix. T_s can be composed as $T_s = V \Lambda V^T$, where Λ is a diagonal matrix with the eigenvalues of T_s, which are the same as the eigenvalues of T and V is an orthogonal matrix ($VV^T = Id$) which columns are the eigenvectors of T_s. Then,

$$\begin{aligned} T &= D^{-1/2} T_s D^{1/2} \\ &= D^{-1/2} V \Lambda V^T D^{1/2} \\ &= \Psi \Lambda \Phi^T \end{aligned} \tag{1}$$

with $\Psi = D^{-1/2} V$ and $\Phi = D^{1/2} V$.
