# Supplementary material for: Random walk informed heterogeneity detection reveals how the lymph node conduit network influences T cells collective exploration behavior
Source: PLoS Comput Biol. 2023 May 24;19(5):e1011168. doi: 10.1371/journal.pcbi.1011168 (PMC10243635; doi:10.1371/journal.pcbi.1011168)
Supplement: S2 Text — (PDF) [file pcbi.1011168.s002.pdf]

S2 Text Numerical approximation of the spectral decomposition for the large networks

The computation of the full spectral decomposition of a symmetric matrix A of size $N \times N$ requires $O(N^3)$ floating point operations and $O(N^2)$ coefficients to store, which becomes intractable even for medium-sized N. In particular, this algorithm fails for the **LNCN** dataset where $N = 200,000$.

However approximating T^t by $\widehat{T}_k^t = V_k \Lambda_k^t V_k^T$ only requires the computation of the truncated decomposition $V_k \Lambda_k V_k^T$ where V_k is of size $N \times k$ and contains the eigenvectors associated to the k largest eigenvalues and $\Lambda_k = \text{diag}(\lambda_0, \dots, \lambda_k)$ is a diagonal matrix of size $k \times k$. This observation can be leverage to implement spectral decomposition algorithm that are fast provided that $k \ll N$.

We used the *eigsh* function from the *scipy* library which implements a Lanczos method [1, 2]. This method is iterative and mainly needs the computation of k matrix-vector products and an orthogonalization step of complexity $O(k^2 N)$. For general matrices, the cost of k matrix-vector products is $O(kN^2)$, but for sparse matrices, this complexity reduces to $O(kLN)$ where L is the average number of non-zero coefficients in each row. The total complexity of the Lanczos method for sparse matrices is therefore bounded by $O(k^2 N + kLN)$. For the **LNCN** dataset, the matrix T_s contains roughly $L = 3$ non-zeros per row, leading to a very efficient diagonalization procedure.

References

[1] Gene H Golub and Charles F Van Loan. *Matrix computations.* JHU press, 2013.

[2] Richard B Lehoucq, Danny C Sorensen, and Chao Yang. *ARPACK users' guide: solution of large-scale eigenvalue problems with implicitly restarted Arnoldi methods.* SIAM, 1998.
