# Supplementary material for: Random walk informed heterogeneity detection reveals how the lymph node conduit network influences T cells collective exploration behavior
Source: PLoS Comput Biol. 2023 May 24;19(5):e1011168. doi: 10.1371/journal.pcbi.1011168 (PMC10243635; doi:10.1371/journal.pcbi.1011168)
Supplement: S3 Text — (PDF) [file pcbi.1011168.s003.pdf]

S3 Text Estimation of the error of the approximation of the spectral decomposition of the transition matrix

In this section we describe the computation of the approximation error between T^t to $\widehat{T}_k^t$ as defined in the paragraph **Approximation** of the Materials and Methods section of the main part of the paper.

Let A be a matrix of size $M \times N$, its spectral norm $\|A\|_{2,2}$ is given by

$$\|A\|_{2,2} = \sup_{x \in \mathbb{R}^N, \|x\|_2 = 1} \|Ax\|_2. \quad (1)$$

Importantly, the spectral norm of a matrix is equal to its largest singular value i.e. $\|A\|_{2,2} = \sigma_1(A)$.

This norm induces the spectral distance $\|A - B\|_{2,2}$ between two matrices of size $M \times N$ and measures the maximal error that can be made between the two vectors Ax and Bx with $x \in \mathbb{R}^N$.

Let $\Delta = T^t - \widehat{T}_k^t$. The computation of $\|\Delta\|_{2,2}$ requires the computation of $\sigma_1(A) = \sqrt{\lambda_1(\Delta^T \Delta)}$, with $\lambda_1(\Delta^T \Delta)$ referring to the largest eigenvalue of $\Delta^T \Delta$.

Large graphs prevents the matrix $\Delta^T \Delta$ to be stored in memory, we therefore need an indirect way of computing its largest eigenvalue. The power iteration method [1, Section 8.2.1 p406] computes the largest eigenvalue of a given matrix using only its matrix-vector products, which makes it suitable for large scale graphs.

We implemented the power method on $\Delta^T \Delta$ through the two the matrix-vector products with Δ^T and Δ. This products are made of:

- t matrix-vector products with the sparse matrix T and T^T. For a matrix of LN non-zero coefficients, this step costs $O(tLN)$ operations.

- matrix-vector products with $\widehat{T}_k^t = \Psi \Lambda_k^t \Phi^T$ and $(\widehat{T}_k^t)^T = \Phi \Lambda_k^t \Psi^T$ which both cost $O(kN)$ operations.

For the **LNCN** dataset, the matrix T contains roughly $L = 3$ non-zeros per row, leading to a fast computation of $\|\Delta\|_{2,2}$.

We define the relative error as $\frac{\|\Delta\|_{2,2}}{\|T^t\|_{2,2}}$, with $\|T^t\|_{2,2}$ also computed with power iteration method.

References

[1] Gene H Golub and Charles F Van Loan. *Matrix computations*. JHU press, 2013.
