# Supplementary material for: Random walk informed heterogeneity detection reveals how the lymph node conduit network influences T cells collective exploration behavior
Source: PLoS Comput Biol. 2023 May 24;19(5):e1011168. doi: 10.1371/journal.pcbi.1011168 (PMC10243635; doi:10.1371/journal.pcbi.1011168)
Supplement: S4 Text — (PDF) [file pcbi.1011168.s004.pdf]

S4 Text Relaxation time calculation

The relaxation time τ is defined as the time for which
$|p(j,t|i) - p(j,t=\infty)| \leq e^{-1}$
According to https://www.stat.berkeley.edu/∼aldous/RWG/Chap4.pdf,
$|p(j,t|i) - p(j,t=\infty)| \leq \lambda_1^t$
Thus the condition
$|p(j,t|i) - p(j,t=\infty)| = e^{-\frac{t}{\tau}} = \lambda_1^t$
gives

$$\begin{aligned} e^{-\frac{t}{\tau}} &= \lambda_1^t \\ -\frac{t}{\tau} &= t\log(1-(1-\lambda_1)) \\ -\frac{t}{\tau} &\underset{\lambda_1\to 1}{\sim} -t(1-\lambda_1) \\ \tau &\underset{\lambda_1\to 1}{\sim} \frac{1}{1-\lambda_1} \end{aligned} \tag{1}$$

Thus, $< p_{in} >_C (t) \underset{t\to+\infty}{\longrightarrow} \frac{1}{|C|}\frac{\sum_{i\in C} d_i}{d_{tot}}$

Similarly $< p_{out} >_C (t) \underset{t\to+\infty}{\longrightarrow} \frac{1}{|\bar{C}|}\frac{\sum_{i\in \bar{C}} d_i}{d_{tot}}$
