# Supplementary material for: Random walk informed heterogeneity detection reveals how the lymph node conduit network influences T cells collective exploration behavior
Source: PLoS Comput Biol. 2023 May 24;19(5):e1011168. doi: 10.1371/journal.pcbi.1011168 (PMC10243635; doi:10.1371/journal.pcbi.1011168)
Supplement: S5 Text — (PDF) [file pcbi.1011168.s005.pdf]

S5 Text Features of $< p_{in} >_C$ and $< p_{out} >_C$

- $< p_{in} >_C (t) = < p_{out} >_C (t)$ for regular graphs

$$< p_{in} >_C (t) = \frac{1}{|C||\bar{C}|} \sum_{i \in C} \sum_{j \in \bar{C}} \sum_{k}^{K} \psi_k(j) \lambda_k^t \phi_k(i) \quad (1)$$

$$< p_{out} >_C (t) = \frac{1}{|C||\bar{C}|} \sum_{i \in C} \sum_{j \in \bar{C}} \sum_{k}^{K} \psi_k(i) \lambda_k^t \phi_k(j) \quad (2)$$

By definition of $\phi_k(i)$ and $\psi_k(i)$, $\forall i, \phi_k(i) = \frac{\psi_k(i)}{d_i}$,

$$< p_{in} >_C (t) = \frac{1}{|C||\bar{C}|} \sum_{i \in C} \sum_{j \in \bar{C}} \sum_{k}^{K} \psi_k(j) \lambda_k^t \frac{\psi_k(i)}{d_i} \quad (3)$$

and

$$< p_{out} >_C (t) = \frac{1}{|C||\bar{C}|} \sum_{i \in C} \sum_{j \in \bar{C}} \sum_{k}^{K} \psi_k(i) \lambda_k^t \frac{\psi_k(j)}{d_j} \quad (4)$$

Thus if the graph is regular $\forall i, j \ d_i = d_j = d$

Then $< p_{in} >_C (t) =< p_{out} >_C (t)$

- $< p_{in} >_C (t)$ and $< p_{out} >_C (t)$ converge to respectively the relative volume of C and $\bar{C}$ when $t \longrightarrow \infty$

Given that $1 = |\lambda_0| > |\lambda_1| > ... > |\lambda_N| > 0$

$< p_{in} >_C (t) \underset{t \to +\infty}{\longrightarrow} \frac{1}{|C||\bar{C}|} \sum_{i \in C} \phi_0(i) \sum_{j \in \bar{C}} \psi_0(j)$

Which can be re-writen

$\frac{1}{|C||\bar{C}|} \sum_{i \in C} \phi_0(i) \sum_{j \in \bar{C}} \psi_0(j) = \frac{1}{|C||\bar{C}|} \frac{1}{d_{tot}} \sum_{i \in C} d_i \sum_{i \in |\bar{C}|} 1 = \frac{1}{|C|} \frac{\sum_{i \in C} d_i}{d_{tot}}$

Thus, $< p_{in} >_C (t) \underset{t \to +\infty}{\longrightarrow} \frac{1}{|C|} \frac{\sum_{i \in C} d_i}{d_{tot}}$

Similarly $< p_{out} >_C (t) \underset{t \to +\infty}{\longrightarrow} \frac{1}{|\bar{C}|} \frac{\sum_{i \in \bar{C}} d_i}{d_{tot}}$
