# Supplementary material for: Random walk informed heterogeneity detection reveals how the lymph node conduit network influences T cells collective exploration behavior
Source: PLoS Comput Biol. 2023 May 24;19(5):e1011168. doi: 10.1371/journal.pcbi.1011168 (PMC10243635; doi:10.1371/journal.pcbi.1011168)
Supplement: S1 Fig — Relative spectral norm between the exact transition matrix and the approximated one with truncation at k = 2000 first eigenvalues, decaying with time steps. Insert: log scale representation. (PDF) [file pcbi.1011168.s006.pdf]

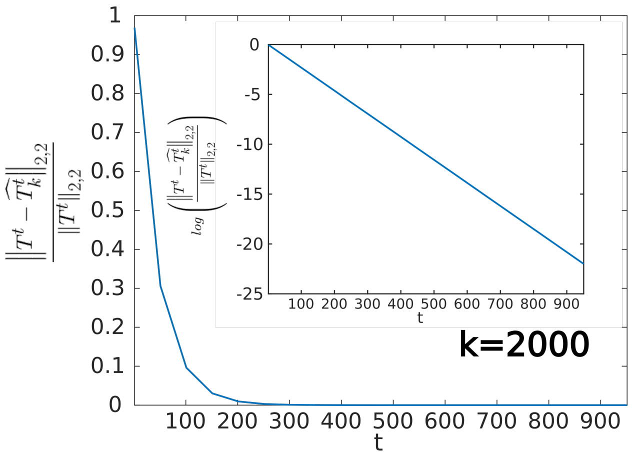

S1 Fig Relative error estimation of the approximated transition matrix for the LNCN Relative spectral norm between the exact transition matrix and the approximated one with truncation at k=2000 first eigenvalues, decaying with time steps. Insert: log scale representation.
