# Supplementary material for: Random walk informed heterogeneity detection reveals how the lymph node conduit network influences T cells collective exploration behavior
Source: PLoS Comput Biol. 2023 May 24;19(5):e1011168. doi: 10.1371/journal.pcbi.1011168 (PMC10243635; doi:10.1371/journal.pcbi.1011168)
Supplement: S2 Fig — A-D: small toy network that exemplifies the difference between diffusion communities and Infomap. In this network, Infomap (D) detects only one community, that encompasses all the nodes. In the workflow we propose, the diffusion communities are made after choosing k, the number of communities when applying the k-means algorithm on the diffusion coordinates, for t = τ = 1. k is a parameter one needs to adjust, which allows to tune the resolution. From k = 2 to k = 4 (A-C) the diffusion communities are more and more precise. For k = 4, the communities illustrate well the interpretation of diffusion communities as groups of nodes from which random walkers follow close trajectories. E: Infomap algorithm applied on the LNCN yields 2 clusters, with codelength 5.75. Diffusion communities, as presented in this study allows to detect higher resolution communities by chosing k = 100. (PDF) [file pcbi.1011168.s007.pdf]

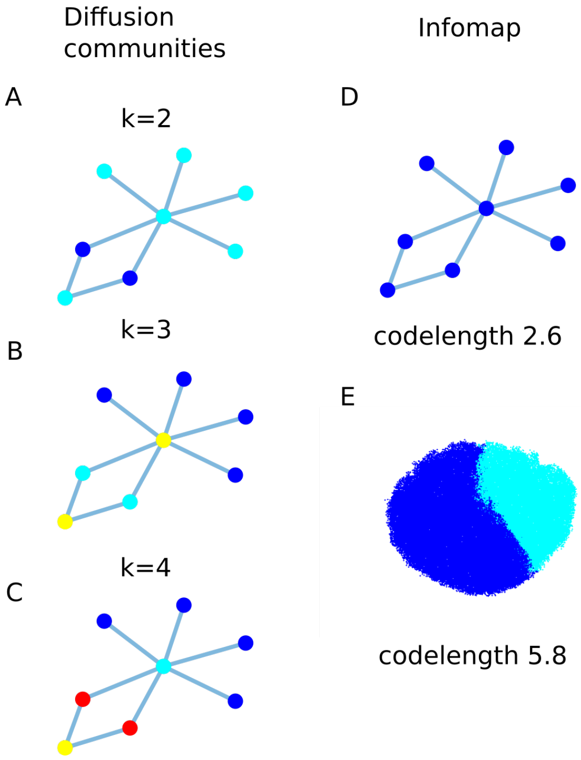

S2 Fig This figure shows how diffusion communities differ from Infomap algorithm[1], a state-of-the-art community detection method based on random walk. A-D: small toy network that exemplifies the difference between diffusion communities and Infomap. In this network, Infomap (D) detects only one community, that encompasses all the nodes. In the workflow we propose, the diffusion communities are made after choosing k, the number of communities when applying the k-means algorithm on the diffusion coordinates, for $t = \tau = 1$. k is a parameter one needs to adjust, which allows to tune the resolution. From $k = 2$ to $k = 4$ (A-C) the diffusion communities are more and more precise. For $k = 4$, the communities illustrate well the interpretation of diffusion communities as groups of nodes from which random walkers follow close trajectories. E: Infomap algorithm applied on the **LNCN** yields 2 clusters, with codelength 5.75. Diffusion communities, as presented in this study allows to detect higher resolution communities by chosing $k = 100$

References

[1] Martin Rosvall and Carl T Bergstrom. “Maps of random walks on complex networks reveal community structure”. In: *Proceedings of the national academy of sciences* 105.4 (2008), pp. 1118–1123.
