# Supplementary material for: Random walk informed heterogeneity detection reveals how the lymph node conduit network influences T cells collective exploration behavior
Source: PLoS Comput Biol. 2023 May 24;19(5):e1011168. doi: 10.1371/journal.pcbi.1011168 (PMC10243635; doi:10.1371/journal.pcbi.1011168)
Supplement: S3 Fig — For the null networks, the networks are represented as graphs, the embedding being the default layout of Matlab. On the contrary, the LNCN is represented with the 3D coordinates of its nodes. For each network, for each community we show the values as colormap of the mean degree < d >C, the mean entry and exit probabilities < pin >C (τ) and < pout >C (τ) at relaxation time, as well as the Cheeger mixing value < h >C. (PDF) [file pcbi.1011168.s008.pdf]

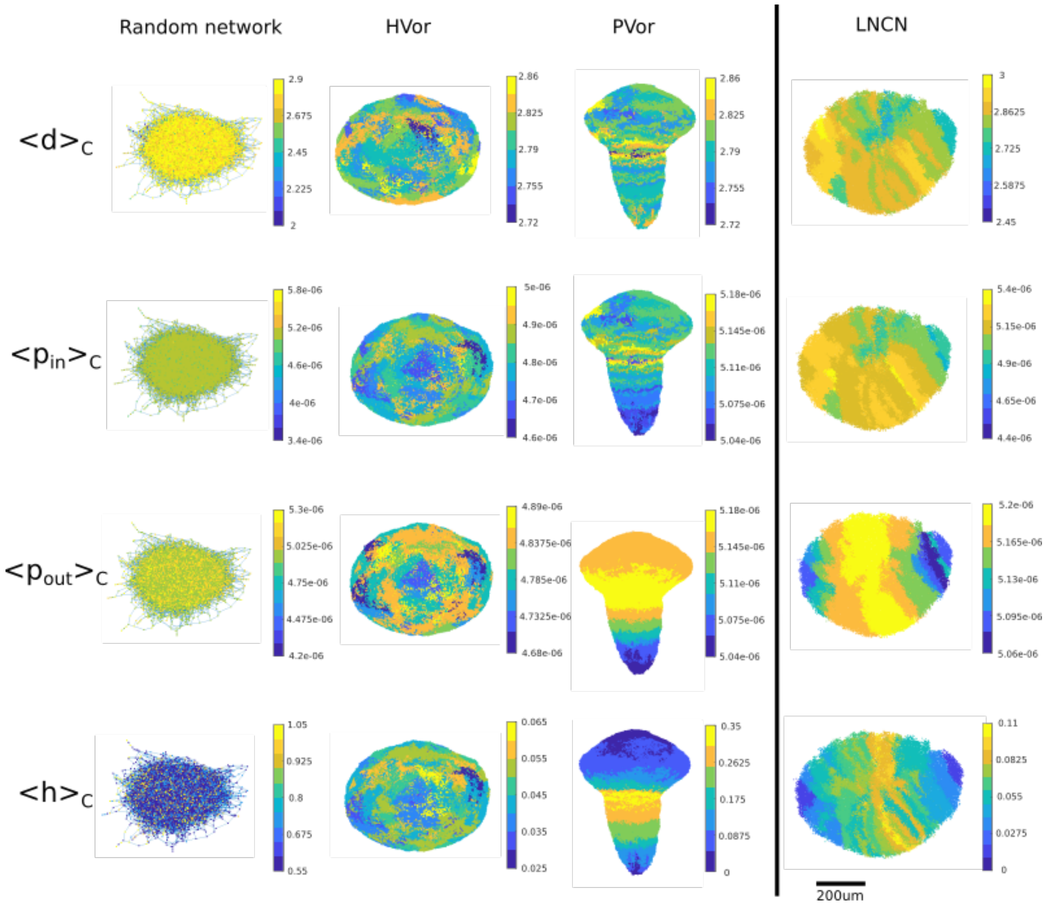

S3 Fig Features colormaps for the diffusion communities in the null networks Random network, HVor, PVor, and for the LNCN. For the null networks, the networks are represented as graphs, the embedding being the default layout of Matlab. On the contrary, the **LNCN** is represented with the 3D coordinates of its nodes. For each network, for each community we show the values as colormap of the mean degree $< d >_C$, the mean entry and exit probabilities $< p_{in} >_C (\tau)$ and $< p_{out} >_C (\tau)$ at relaxation time, as well as the Cheeger mixing value $< h >_C$.
