# Supplementary material for: Random walk informed heterogeneity detection reveals how the lymph node conduit network influences T cells collective exploration behavior
Source: PLoS Comput Biol. 2023 May 24;19(5):e1011168. doi: 10.1371/journal.pcbi.1011168 (PMC10243635; doi:10.1371/journal.pcbi.1011168)
Supplement: Attachment — Submitted filename: response_reviewers.pdf [file pcbi.1011168.s009.pdf]

Dear Editors,

Thank you for the opportunity to revise our manuscript. We describe below our answers to the reviewers. We have performed major revisions to our initial manuscript.

The aim of our study is to evaluate the influence of the lymph node conduit network connectivity patterns on the collective exploration behaviors of T cells. To study this question, we have developed an original pipeline to identify random walk related heterogeneities in large networks. To establish the significance of our results, we have first reorganized our analysis and compared the lymph node conduit network with null models of similar sizes. We have then decided to focus on two descriptive features, the Cheeger mixing index and the entry and exit probability statistics. Finally, we highlighted more precisely the consequences of the results of our analysis on the T cells behavior within the lymph node. Specifically, we demonstrate that:

1) The structure of the lymph node conduit network is spatially coherent (nodes that are well connected by random walks are also close topological neighbors),

2) Despite the quasi-regularity of the network (most nodes being degree 3), we can distinguish regions in the networks (communities of nodes) with different entry and exit probabilities

3) At the relevant biological time scale, there are a few communities that stand out by their mean entry and exit probabilities, they are located on the extreme edges of the longest axis of the lymph node, and one community putatively at the medulla, which have especially low entry and exit probabilities, which we call in the revised manuscript “remote regions”

4) However, overall, the LNCN does not provide directional cues, in the sense of attracting cells into a specific region, where the dendritic cells could sit preferably. Indeed the remote regions, although they might be interesting if mapped more precisely by future anatomical and functional study, occupy very restricted volume of the T-cell zone. This indicates that, despite variations in the connectivity patterns, the overall network structure promotes **an exhaustive unbiased collective exploration**.

We hope that the revised version of our manuscript will meet the standards of *PLOS Computational Biology*, and that you will consider this work for publication in your journal.

Thank you for your efforts, we look forward to hearing from you soon.

Sincerely,

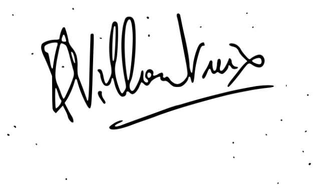

Paul Villoutreix

Reviewer #1:

Song et al. develops a method to assess heterogeneous networks using the Cheeger mixing index. The authors then develop both 2D and 3D toy networks and use the Cheeger mixing index to compare features of network connectedness to data from staining of lymph nodes obtained from Kelch et al 2019.

The use of new quantitative measures to determine biological features of networks such as LNs is novel and interesting.

We thank reviewer 1 for taking the time to read our manuscript in depth and for her positive assessment of our work. Based on Reviewer 1 and 2 assessment of our manuscript, we performed major revisions to our text. We describe below our answers and changes.

However, there are several issues with the application of the Cheeger value both to model networks and biological networks. For example, while Fig 4A shows interesting differences between the HCN and PCN, the Cheeger mixing index (Fig 4F) shows similar values for HCN and PCN. Although the Cheeger value can distinguish between random networks and HCN and PCN, it's not clear how useful this measure is likely to be to actually distinguish complex networks such as biological networks of LNs. Either larger planar networks should be tested to show that Cheeger mixing value can be useful to distinguish or discussion of how Cheeger is meaningful needs to be better explained. Otherwise, it's not clear how Cheeger value is useful.

The goal of our approach consists in characterizing the influence of global network connectivity on the collective exploration behavior derived from a random walk on the network. We start from the adjacency matrix of the network. From it, we derive 'diffusion coordinates' for each node of the network; the distances between any pair of nodes computed with these coordinates reflect how accessible these nodes are on average over a large number of random walkers.

We then use these diffusion coordinates to compute communities using the k-means algorithm. The Cheeger mixing index is computed for each community to evaluate if these communities are well connected within the original network. Its average value over all communities for a given network can then be used as a descriptive feature of the entire network and used to compare various network topologies.

To clarify our approach, in the revised version of the manuscript, we decided to focus on the comparison between the lymph node conduit network (LNCN) and comparable null models. Comparable null models are networks with similar number of nodes and average degree. **These null models characteristics are listed in Table 1, and their respective average Cheeger mixing values can be found in Figure 3F.**

Formally, the Cheeger mixing value measures to what extent nodes which are well connected by diffusion paths (numerous short paths) are also neighbors topologically (a few nodes apart). Such measure must be low in planar (the equivalent is mesh-like in 3D embedding) networks in which the edges don't cross and thus offer no shortcut between topologically distant nodes. On the contrary, a random network should have a high Cheeger mixing value. In order to evaluate if the Cheeger mixing value of the LNCN should be considered high or low, we compared it to a series of other large networks. These networks are obtained from increasing randomization of a

“planar”/mesh-like large network, the HVor, which is a Voronoi mesh. As expected, the average Cheeger index increases for networks with an increasing number of randomly rewired edges. Compared to these networks, the LNCN stands with the lowest values of Cheeger mixing, thus displays high spatial coherence, i.e. no shortcuts.

Previously, the small toy models results were intended to give more intuition of the Cheeger mixing values tendencies using known classes of network: planar, geometric and random, with respectively low, mild and high values. This point is now made clearer in the revised manuscript as the increasing values of Cheeger mixing index as the randomization of HVor increases. The small toy models were removed from the revised manuscript, so that the only networks being presented all are large and have the same mean degree as the LNCN to avoid confusion.

An additional problem with whether Cheeger value can distinguish between biologically distinct networks also came with the 3D toy networks. In Fig 5J, the Cheeger value looks similar between LNs, PVor, and HVor. Thus, it is difficult to assess whether Cheeger can shed any new light on network properties, either toy networks or biological networks. As the manuscript stands, it is not clear Cheeger value will give meaningful interpretation to 3D networks.

In the first version of the manuscript, the emphasis we put on HCN and PCN which can be drawn as planar networks in 2D was misleading, maybe falsely inducing a distinction between 2D and 3D networks. We treat all networks as graphs: nodes connected by edges, without referring to any spatial embedding.

We added new models that are more comparable to the LNCN. Particularly, HVor is a Voronoi mesh, displaying an average Cheeger index value very similar to LNCN. The fact that the Cheeger mixing is as low in the LNCN as in the HVor and lower than PVor is precisely what sheds light on the network properties of the LNCN. Indeed HVor and PVor are representatives of networks with very low expected Cheeger mixing values due to their construction rules. Thus the fact that the value for LNCN is comparable to these indicates that the LNCN is highly spatially coherent.

The comparison between the Cheeger index average value of the LNCN and HVor and PVor is displayed on Figure 3 F.

Of even more significant concern is the applicability of the Cheeger mixing value to biological data such as lymph nodes. To utilize biological data, reproducibility must be considered. The authors already point out a significant and problematic limitation of the use of Kelch et al. data in that only one lymph node is stained. To base quantitative measurements on a single data point is not acceptable as reliable biological data.

The approach and arguments that we propose rely on the fact that we have access to the network in its entirety. As far as we know, the Kelch et al. dataset is a unique dataset that provides a complete reconstruction of the whole lymph node conduits network. Novkovic et al, 2020, on the other hand, provides a slide of T cell zone of 300x300x30um and not the full LN conduits network (850 x 750 x 900 um). Thus the Kelch et al. dataset provides a rare opportunity to conduct these analyses at a much larger scale than previously done. When additional instances of LNCN are made available our pipeline can readily be applied on new samples. Furthermore, the strength of our study relies on the fact that we find that features of the LN are significantly different from a variety of null models.

Furthermore, there are multiple types of stainings utilized in Kelch et al, including WGA staining which stains the full lymph node, including B cell follicle, germinal centers, T cell zone, and medullary regions. These areas are distinguished with antibody staining but it is not clear from Song et al. whether this manuscript only analyzes the T cell zone. As the T cells are only migrating in the T cell zone, taking into account the conduit network based on WGA staining of GCs and B cell follicles would not be appropriate and in fact, may present inaccurate information about the conduit network in the T cell zone. Thus, it is important to specify precisely what zones and what stainings are used in this manuscript to analyze LN networks in this manuscript.

Thank you for pointing out this ambiguity. The LNCN as shared with us by Kelch et al only has the T cell zone.

- Medulla and subcapsular sinus were removed using the fact they are completely filled with WGA staining, contrarily to the T cell zone conduits which are thinner and thus are not completely filled.

- B cell follicles including potential germinal centers were removed manually by Kelch et al.

- Germinal centers are rare in the popliteal lymph node of unstimulated mice

We added some precisions in the manuscript lines 212-214 of the revised manuscript.

Additionally, it will be important to use additional networks, such as the T cell zone stainings for FRCs published in Novkovic Plos Biol 2016 to validate the LNCN networks and not rely on only a single lymph node.

We chose not to include the data from Novkovic et al. mainly because of the fact that their reconstruction is only a slice of the T cells zone and is thus not comparable to the Kelch et al. dataset we apply our global scale analysis on. More specifically, our approach relies on the entire network connectivity, and using only a subpart of the network would generate biases and artifacts in our analysis. The reason is that by setting aside a subset of the network (everything that is outside the slice of the T cells zone), we would lose the effect and influence of all the possible random walk paths moving through that region. Additionally, in the specific case of the publication Novkovic 2016, the network reconstructed is the FRC network, which differs considerably from the conduits network. The reconstruction of the FRC network relies on modeling the center of mass of the cells as nodes and each protrusion as an edge. This reconstruction method yields a network with very different characteristics from the conduit network, as the authors themselves show in Novkovic Cell Reports 2020. For us, whether the T cells follow the collagen of the conduits network or the FRCs network remains an open question. In our study we use the working hypothesis that the T cells follow the conduits network. It seemed to us a more interesting choice because the data is available for a whole lymph node T cells region. **We made this choice appear more clearly in the discussion part lines 397-408**, thank you for raising this point. We emphasize that when such whole lymph node T cell zone data becomes available for the FRCs network, it will be interesting to apply our pipeline to it for comparison.

One of the major conclusions from the manuscript is that T cell motility is random. This random motility has already been shown previously by analysis of experimental T cell tracks as well as

computationally. In the discussion, it was not clearly indicated how using Cheeger value sheds new light on how LN connectivity affects T cell search for lymph nodes. It will be important to discuss the novel contribution of these new analysis on T cell behavior and how it likely impacts overall immune responses.

Indeed, we had not made clear enough in the first version of the manuscript that experimental T cells tracks have already been characterized and shown to display random walk characteristics. **We made this point clearer and how our study of the network connectivity fits with respect to these studies in the introduction of the revised manuscript lines 22-30**. Despite the fact that the T cells tracks are known to have random walk characteristics, this behavior can result from different complex factors that include both internal and environmental cues for the cells. Among these cues, the fact that these T cells were shown to follow the LNCN network is the particular aspect that we address : how does the network connectivity influence the motility of T cells at the whole T-cell zone scale?

The answers that we provide were also not clearly enough stated so we re-organized the manuscript to make them appear more clearly.

The first conclusion is that the LNCN is spatially coherent : nodes that are well connected by random walks are also close topological neighbors. This is the point we made by computing the average Cheeger index, and comparing the value with a series of null networks. **This point is made by Figure3 and section "The Cheeger mixing index compared between LNCN and null models show that the LNCN is spatially coherent" in the revised manuscript.** The other conclusions we made are not derived from the average Cheeger index but from the measure of mean entry and exit probabilities of the diffusion communities.

The second conclusion we draw is that despite the quasi-regularity of the network (most nodes being degree 3), we can distinguish regions in the networks (communities of nodes) with different entry and exit probabilities. These differences are significant compared to null models, such as a random network of same size and same mean degree, and the HVor, which has not only same size and same mean degree as the LNCN but also the same average Cheeger index. **This point is made by Figure4 and section "<pin>C and <pout>C comparison between the LNCN and null models shows that the LNCN is significantly heterogeneous" in the revised manuscript.**

The third conclusion is that at the relevant biological time scale, there are a few communities that stand out by their mean entry and exit probabilities. They are located on the extreme edges of the longest axis of the lymph node, and one community putatively at the medulla, which have especially low entry and exit probabilities, which we call in the revised manuscript "remote regions". The rest of the volume of the T-cell zone appears uniform. **This point is made by Figure5 and section "The LNCN displays remote regions at its poles and next to the medulla while being homogeneous on most of its total volume, promoting overall uniform collective exploration behavior" in the revised manuscript.**

Our study indicates that the LNCN does not provide directional cues, in the sense of attracting cells into a specific region, where the dendritic cells could sit preferably. Indeed the remote regions, although they might be interesting if mapped more precisely by future anatomical and functional study, occupy a very restricted volume of the T-cell zone. This indicates that, despite variations in the connectivity patterns, the overall network structure promotes an exhaustive unbiased collective exploration.

Reviewer #2: report on manuscript D-22-01540

First and foremost, I want to apologize with the authors for the delay in the delivery of my report. Unfortunately, several issues (international relocation, extra caring duties, and administrative deadlines) have reduced considerably my ability to deliver this report on time.

In this manuscript, the authors present a characterization of the lymph node conduits network (hereafter LNCN) to assess whether its structure optimizes exploration by making some regions more accessible than others. To achieve such a goal, the authors compare the properties (the Cheeger index, h, and the enter -- p_{in} -- and exit -- p_{out} -- probabilities) of the communities of the LNCN dual network existing in the so-called diffusion space. The diffusion space is the space where the nodes' relative positions quantify how often two nodes tend to belong to the path made by distinct random walkers. The authors extract the communities of the dual LNCN network by using the k-means algorithm on the position of the nodes in the diffusion space. Finally, to measure the diffusion properties of the LNCN network, the authors make a comparison of the values of h, p_{in}, and p_{out} computed on the latter, and the same indicators computed for several benchmark networks. The authors have found that although the properties of the LNCN network display a certain spatial regularity, some of its regions seems to promote exploration more.

The application of network science and stochastic processes tools for the analysis of biological systems has proven very useful for scientists working in biological complex systems. The present manuscript poses an interesting question but, I have several concerns about the methodology used to address it. Therefore, my recommendation is that the authors address these concerns and submit a revised version of the manuscript. In the following, I will provide a more detailed explanation of the manuscript's main flaws, together with some suggestions to help the authors addressing them.

We thank Reviewer 2 for their time reviewing this paper and their clear summary of our contributions. Based on Reviewer 1 and Reviewer 2 comments, we made major revisions to our initial manuscript that we detail below.

MAIN ISSUES

-- One of the manuscript's pivotal points is to group nodes not according to their topological features (i.e., physical connections) but, rather, according to their diffusion properties. As the goal of the manuscript is to assess how optimized for exploration the structure of the LNCN is, using the diffusion space seems quite natural. However, from a network perspective I have found the decision to project the nodes onto a diffusion space first, and their assignment to a fixed number of communities -- via the k-mean algorithm, -- then, quite intricate (maybe I have missed something).

This is exactly the approach that we propose. As noted by the reviewer, the use of the diffusion space is quite natural to characterize the propensity of nodes to belong to certain paths by

random walkers, and the associated Euclidean distance provides an adapted framework to compare nodes. We then use the simple k-means algorithm to cluster nodes with similar diffusion properties. Nodes that belong to the same clusters if they are well interconnected by random walks paths. This clustering doesn't necessarily respect the initial connectivity of the network : nodes of the same diffusion community might be scattered in the network in the sense of being many nodes apart on shortest paths connecting them. This potential difference between relatedness in the diffusion space (through random walk paths) and relatedness in the original network (through shortest paths) is measured by the cheeger index, which enables to characterize the spatial coherence of these communities.

Choosing k of k-means introduces a parameter to adjust. However, here we use the clusters obtained by k-means as a way to provide a coarse-grained description of the network, varying the value of k will vary the resolution at which we observe the diffusion properties of the network. For the lymph node conduit network (LNCN) for example, we chose to use k = 100 as a good tradeoff between a precise description of the structure and a number of clusters that can still be explored visually. We found this approach to be quite natural given our focus on the random walk properties of the network at region-wise scale.

In addition to the parameter k in k-means, we have a second parameter, t, which encodes the length of the random walk paths that we use to compute the diffusion coordinates. This parameter t will enable us to consider random walks of varying lengths. Different values of this parameter will lead to different descriptions in the diffusion space. Using this parameter, we can therefore identify heterogeneities at short, medium and long time scale. The diffusion communities were made with t=tau, the relaxation time of each network, so that they are comparable between them.

To clarify the fact that we are using the number of communities and the time parameter as two ways of controlling the scale at which we observe the network, **we added a sentence at lines 63-66 in the introduction of the revised manuscript.**

Please note that the Louvain algorithm applied on the original network gives 75 communities, which is in the same order of magnitude as k=100.

There exist, in fact, several methods based on random walks to extract the community structure of a network which perform really well in case of "flow networks" like the LNCN. The most famous of these methods is the so-called Infomap algorithm:

https://www.pnas.org/doi/full/10.1073/pnas.0706851105

One of its authors (Martin Rosvall) maintains a very nice website where, beside information on the algorithm, one can run directly the infomap on its own network

https://www.mapequation.org/

I strongly recommend to, at least, replicate the analysis proposed in this manuscript using the community structure extracted with Infomap directly on the structural network. This step will remove the problem of arbitrarily fixing the number of communities (which does not make much sense to me), as well as reduce the biases on the interpretation of the results found.

We thank the reviewer for bringing to our attention this very relevant method. We computed it on our LNCN network and provided a visualization of the communities obtained. A discussion of the communities detected by infomap compared to our pipeline is presented on **S2 Fig. E.** Particularly, the use of infomap on the lymph led to only two communities in the Lymph Node. The communities obtained do not seem to be correlated to any of the anatomical regions of the lymph node. This suggests that the modularity is low and the two communities include around 100,000 nodes each. Moreover the comparison of the communities computed on a small toy model using either the infomap algorithm S2 Fig. D or our diffusion communities pipeline with varying value of k highlights the differences in the approaches and the interest of having k as a varying parameter to tune the resolution of the communities.

More theoretically, even though the infomap method is very relevant considering the question of identifying communities based on their random walk behavior, we noted that the approach considered random walks of various lengths, including infinite sequences. We know that the probability of presence derived from a random walk at an infinite time scale is proportional to their degree. The networks that we considered are highly regular in the sense that almost all nodes have degree 3. Therefore, considering infinite sequences of random walk will 'discard' interesting heterogeneities. Moreover, the processes that we are considering are at shorter time scales corresponding to relevant biological times. We thought it more useful, in the context of our application, which is the exploration of T-cells by Lymph Nodes, to be able to analyze the behavior of random walks at intermediary time scales and highlight this way the heterogeneities of the network. Moreover, as explained earlier, in addition to the time scale that we can vary, we used the value k of the number of communities to change the resolution at which we observe our system.

-- Another pivotal point of the manuscript is to compare the diffusion properties of the LNCN network with some benchmark models. I am quite sympathetic with this approach but not entirely satisfied with the criteria used to select which these benchmarks and their properties.

We thank the reviewer for the suggestion, we performed major updates to the comparison to null models. First the large null models have a similar number of nodes and degree than the original LNCN, second, we now include the configuration model in our comparison, and third, we decided to remove the comparison to the small toy models from the main text for clarity.

More specifically:

** The first benchmark I would have considered, would have been a randomized counterpart of the LNCN based on the so-called configuration model. Basically, one keeps the number of nodes, the number of edges, and the degree sequence (i.e., the list of the degrees of each node) of the original network and rewire the connections uniformly at random. There exist a

quite efficient algorithm (beside the one available in NetworkX) which generates such type of networks:

https://epubs.siam.org/doi/10.1137/16M1087175

Thank you for this suggestion. In the revised manuscript we now have a comparison with a the null model which is called "Random network" and which uses the configuration model algorithm. We have tried to use the configuration model algorithm from NetworkX library to directly generate a network with the same sequence of degrees as the LNCN. However, the algorithm would only yield disconnected graphs. For this reason, we used configuration model algorithm to first generate a regular network of degree 3 of same size as the LNCN and we removed nodes randomly until the mean degree reaches 2.8, like the LNCN while checking that the network remains connected. **The generation process is described in the Datasets subsection lines 232-235**. **The results of its comparison with the LNCN are shown on Figure4E**

** One criterion which is usually kept in consideration when generating benchmark networks is to keep both the number of nodes, N, and the average degree, $\langle k \rangle$, equal to those of the original network. The reason is that usually many features (for instance, related to dynamics like diffusion) are intimately related with the network's size and connectivity. The LNCN network has $N = 192386$ and $\langle k \rangle = 2.86$. However, the benchmark networks do not preserve always these quantities as some networks are considerably smaller $N = 2050$ and more connected $\langle k \rangle \simeq 960$ like in the case of the Random Geometric Graph. Overall, my sensation is that the comparison between the LNCN and the benchmarks is not made under "fair conditions."

Thank you for pointing out this major drawback. We added major revisions to address this. We removed all small models. **We added a series of newly generated networks, all summarized in Table1 of the revised manuscript**. Now, all the network that the LNCN is compared to are about the same size, same mean degree as the LNCN.

** On a less severe extent, it would be good to quantify how "modular" the networks (LNCN and the benchmarks) are. To do so, one could compute for instance the so-called modularity indicator (NetworkX has a built-in function doing it):

10.1016/j.physrep.2009.11.002 (and references therein)

We added the Louvain modularity for all networks in the Table 1.

-- The manuscript does not provide a strong "biological interpretation" of the results found. Perhaps I have missed something but, after reading the manuscript, one question that came to my mind was: "what have we learned from the biological point of view about the LNCN network?" Said in other terms: why it is important to study the diffusion properties of the LNCN, and why what has been found is important/interesting for scientists working with the LNCN? I have also found strange the absence of mentions to already existing results in the Discussion section, as if the results found in this manuscript are disconnected from the existing knowledge on the same topic. Perhaps these points are already covered in the manuscript, but I suggest to make them "shine" more.

Thank you for pointing out this lack of clarity. We re-organized major parts of the manuscript to improve this.

The knowledge gap we intend to fill is : Despite the fact that the T cells tracks are known to have random walk characteristics (based on previous studies with live microscopy tracking of T cells on small sub volumes of the lymph node, without the simultaneous network acquisition), this behavior can result from a variety of complex factors that include both internal and environmental cues for the cells (chemical for instance). Among these cues, the fact that these T cells were shown to follow the LNCN network is the particular aspect that we address : how does the network connectivity influence the motility of T cells at the whole T-cell zone scale?

We added a paragraph in the revised manuscript in the introduction lines 22-32 to make this point more clear.

Then, as for the conclusions of our study, we re-organized the manuscript so that the 3 conclusions are stated respectively in separate sections.

The first conclusion is that the LNCN is spatially coherent : nodes that are well connected by random walks are also close topological neighbors. This is the point we made by computing the average Cheeger index, and comparing the value with a series of null networks. **This point is made by Figure3 and section “The Cheeger mixing index compared between LNCN and null models show that the LNCN is spatially coherent” in the revised manuscript.** The other conclusions we made are not derived from the average Cheeger index but from the measure of mean entry and exit probabilities of the diffusion communities.

The second conclusion we draw is that despite the quasi-regularity of the network (most nodes being degree 3), we can distinguish regions in the networks (communities of nodes) with different entry and exit probabilities. These differences are significant compared to null models, such as a random network of same size and same mean degree, and the HVor, which has not only same size and same mean degree as the LNCN but also the same average Cheeger index. **This point is made by Figure4 and section “<pin>C and <pout>C comparison between the LNCN and null models shows that the LNCN is significantly heterogeneous” in the revised manuscript.**

The third conclusion is that at the relevant biological time scale, there are a few communities that stand out by their mean entry and exit probabilities. They are located on the extreme edges of the longest axis of the lymph node, and one community putatively at the medulla, which have especially low entry and exit probabilities, which we call in the revised manuscript “remote regions”. The rest of the volume of the T-cell zone appears uniform. **This point is made by Figure5 and section “The LNCN displays remote regions at its poles and next to the**

medulla while being homogeneous on most of its total volume, promoting overall uniform collective exploration behavior" in the revised manuscript.

Our study indicates that the LNCN does not provide directional cues, in the sense of attracting cells into a specific region, where the dendritic cells could sit preferably. Indeed the remote regions, although they might be interesting if mapped more precisely by future anatomical and functional study, occupy a restricted volume of the T-cell zone. This indicates that, despite variations in the connectivity patterns, the overall network structure promotes an exhaustive unbiased collective exploration.

-- The presentation style needs to be improved. I believe that a good portion of the "Materials and Methods" section should be moved at the end of the manuscript. The reason is that the current manuscript's organization forces the reader to jump many times back and forth between the methods and the appendices, thus disrupting the reading "flow."

We removed as much technical detail as we could from the "Materials and Methods" to the supplementary information, in order to simplify the reading. We hope it reads better now.

The figures are quite poor as they are very tiny (and, sometimes, almost unreadable) and using a color scheme which does not render well when the manuscript is printed in b/w. Concerning this point, there is a flurry of activity around the proper use of color in scientific communication, as explained very well in this nice paper:

We apologize for the unreadable figures. We re-made most of them to meet the requirements.

https://www.nature.com/articles/s41467-020-19160-7

In general, I recommend to improve the figures by:

** Avoiding using colorscales that are not well rendered in b/w or grayscale (e.g., the jet or rainbow one).

We updated the colorscales of each of the figures for parula colorscale which is perceptually uniform and avoid red-green confusion. We hope that this colorscale renders better.

** Enlarge the figures such that details like the axis and ticks labels in the plots are well readable.

We updated the figures so that the axis and ticks labels are readable.

** Avoiding using 3D display and, whenever possible, favour heatmaps in their place.

We could not avoid all the 3D displays but we uploaded on github the corresponding html files for the Figure 5B-D for 3D interactive visualization.

** In line plots [like those of Fig. 3(k-l)] try to use line styles that allow to discriminate distinct datasets even when the figure is displayed in grayscale.

Because of revisions, we updated the figures, for which we don’t have line plots.

** Add legends to plots if those improve their interpretability.

We added legends to improve interpretability.

** Avoiding using colors like yellow, cyan, and light green on a white background as their contrast is not very high and becomes hard to distinguish them.

We updated the colors of each of the figures.

** Specify always what is displayed on each axis [for instance, improve Fig. S2(a)].

We specified as much as possible what is displayed on each axis.

** Condensate plots whenever possible [for instance, Fig. S4 could be replaced by a single line plot with many lines, each accounting for a distinct case].

We tried to condensate plots as much as possible.

-- In the data and code availability statement, the authors have stated that "All relevant data are available on Zenodo, and code on github repository." However, I have found no trace of any reference to the data/code in the manuscript. Please, fix this problem.

The data and code are now available. The workflow and most of the variables are in the github repository https://github.com/SoleneS/RW_lymph_node.
The spectral decompositions, which are very heavy files are uploaded on Zenodo DOI: 10.5281/zenodo.7681900
https://zenodo.org/record/7681900#.Y_8UOS8w1hF

MINOR ISSUES

-- In the abstract, the LNCN acronym is used without introducing it. Either use the explicit form and remove the acronym or introduce it properly.

We introduced it properly.

-- I believe that the equation appearing in panel E of Fig. 1 is completely useless as the reader is unable to understand its meaning without reading the whole manuscript. I suggest to remove it.

We removed it.

-- On page 5, Equation (2), the sum runs over y but it is not clear what y is.

It was a typo, the sum should run over j all the nodes in the network. We updated the equation.

-- Page 5 line 127, the symbol to describe the number of nodes is N not n. Please correct it.

We changed n to N as it corresponds to the number of nodes.

-- The right way to spell Euclidean is with capital E. Please correct it (there are several instances across the text).

We changed the spelling of euclidean to Euclidean on each instance.

-- Page 6 line 165, there is a missing) in the equation describing the Cheeger coefficient.

We added the missing) to the equation.

-- I suggest to add some reference to the Cheeger mixing coefficient (I had never heard of it insofar).

We added reference 23 for the Cheeger constant.

The Cheeger mixing index is a measure that we define for each community C: number of edges connected a node that belongs to C and a node which does not, divided by the minimum of the volume of C and volume of nodes that don't belong to C. We called this measure Cheeger mixing index because computes this value over all communities possible (all sets of nodes within the network) and takes the minimal value obtained, it coincides with the Cheeger constant.

-- Page 9 line 259. Perhaps I am mistaken but the correct spelling of "sparcity" is "sparsity".

This is correct, we don't have any instance of "sparcity" anymore.

-- Page 12 line 361. There is an extra) in the relation of p_{out}.

We updated the text.

-- Page 12 line 384. Please provide a definition of the diffusion distance.

The diffusion distance is defined in the paragraph “Diffusion coordinates” of the Materials and methods section. The part of the text that the reviewer is referring to has been removed in the new version of the manuscript.

-- Page 15 Fig. 6 caption. The concept of "biological scan time" is not very clear. Please improve the clarity.

We clarify the concept of “biological scan time” **in lines 338-342 of the revised manuscript**

-- Page 17 line 562. Please add a citation to the SciPy library.

We added a citation to the paper “SciPy 1.0: Fundamental Algorithms for Scientific Computing in Python” by Virtanen et al.

-- Page 18 line 573. There is a mention to Eq. (9) of the main manuscript but there are only two equations in the main manuscript. Please clarify this point.

Thank you for noticing this typo, the relevant variable is defined in the paragraph “Approximation” of the Materials and Methods section. We updated the cross reference.

-- The captions and the figures must appear in the same place. However, this is not the case for all the figures of the Appendices. Please fix this.

It is fixed now.

-- Page 18 line 602. The indices of the first term in the left hand side of the equation displayed do not seem correct to me (there are too many i). Perhaps I am wrong but, please, check them.

We updated the indices of this equation, which contained a typo.

-- Page 18 line 603. There is a mistake with the URL displayed there. The correct one is:

https://www.stat.berkeley.edu/~aldous/RWG/Chap4.pdf

We updated with the correct URL.

-- Page 20 line 657. Please state clearly with respect to what the maximum and minimum are computed.

While working on the revised version of the manuscript, we chose to focus on the diffusion properties only and therefore removed the reference to the centralities. This section does not appear anymore.

-- Page 23 line 719. There is a typo (the repeated twice).

We removed the unnecessary ‘the’.

-- Bibliography.

** Ref. 1 . Please provide more information on this entry. To the best of my understanding it refers to the following book:

https://pascal-francis.inist.fr/vibad/index.php?action=getRecordDetail&idt=PASCALZOOLINEINRA8050247635

We updated the reference with the revised version of the book

“Okubo A, Levin SA. Diffusion and ecological problems: modern perspectives. vol. 14. Springer; 2001”

** Ref. 17 . The correct reference for this manuscript is:

https://dl.acm.org/doi/10.5555/2976248.2976368

Please, update the information.

We updated the corresponding reference to the published version.

** Ref. 19 . Please complete the information about this book (missing publisher and edition).

We updated the reference.

** Ref. 20 . Please complete the information about this PhD Thesis. I have found something at:

https://www.theses.fr/2020UNIP7037

This is the correct reference, however, we removed the corresponding reference from the text, since we are not using this 2D toy model anymore.

** Ref. 34 seems a duplicate of Ref. 22 . Please check and, eventually, remove the duplicate entry.

We removed the duplicate.
