# Supplementary material for: Random walk informed heterogeneity detection reveals how the lymph node conduit network influences T cells collective exploration behavior
Source: PLoS Comput Biol. 2023 May 24;19(5):e1011168. doi: 10.1371/journal.pcbi.1011168 (PMC10243635; doi:10.1371/journal.pcbi.1011168)
Supplement: Attachment — Submitted filename: response_reviewers2.pdf [file pcbi.1011168.s010.pdf]

Marseille, April 27th, 2023

Dear Editors,

Thank you for your answer and your planned decision. You can find below a detailed answer to Reviewer 2’s comments.

In addition to the minor revisions suggested by Reviewer 2, we changed for grammatical reasons the expressions 1) “Random walk informed heterogeneities detection” to “Random walk informed heterogeneity detection” and 2) “conduits network” to “conduit network”, throughout the entire article.

We are very honored to have the opportunity to publish our article in PLoS Computational Biology.

Sincerely,

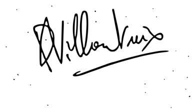

Paul Villoutreix

Group Leader Turing Center for Living Systems
Junior Professor Chair INSERM

Reviewer #2: Second report on manuscript PCOMPBIOL-D-22-01540R1

After reading first the authors' reply to the comments made during the previous round of review, and then the revised version of the manuscript, I have come to the conclusion that the authors have addressed satisfactorily to both reviewers' criticisms. The manuscript is now more solid, clear, and the results are properly highlighted and appealing for scientists working in computational biology (and not), and I thank the authors for their efforts in accommodating the reviewers' suggestions.

In light of this, I can now recommend the acceptance of this manuscript for its publication in PLoS Computational Biology. I have only a couple of minor (cosmetic) remarks that the authors should take care of before final publication.

We thank Reviewer #2 for helping us greatly improve our manuscript.

MINOR ISSUES

-- When introducing the random network benchmark, I believe that beside Ref. 26 it would be advisable to cite the work of Bender and Canfield in which the configuration model was introduced

10.1016/0097-3165(78)90059-6

We added this reference next to where we mention the configuration model.

-- The main idea behind the Cheeger mixing index is very similar to the concept of "participation coefficient" used in the following work of Guimera et al.:

10.1038/nature03288

We added this reference next to where we introduce the Cheeger mixing index, together with the sentence “This measure is similar to the participation coefficient defined in Guimera et al.”

-- Page 6 line 169. The manuscript claims that high values of Cheeger mixing denote that "nodes that belong to the same diffusion community are scattered across the network". However, I believe it is more appropriate to say that high values of Cheeger mixing denote that nodes belonging to the same community do not form contiguous groups as scattered suggests that they are not physically close, which is not necessarily the case.

We updated the sentence and replaced “scattered across the network” by “do not form contiguous groups”

-- Clarity and readability:

** Page 4 line 128. A simpler (and computationally more efficient) way to compute ϕ_{j0} is by replacing the sum at the denominator with a constant equal to twice the total number of edges.

We agree with the reviewer that the sum of the degrees of each of the nodes of the network is equal to twice the total number of edges and updated the manuscript following this suggestion.

** Page 6 line 138. I suggest to remove the dot in the expression of the diffusion distance D as, usually, such a symbol denotes the scalar product and can, thus, generate confusion in the reader.
We updated the equation accordingly.

** Page 6 line 148. When mentioning the k-means algorithm, I believe it would be good to cite the paper where it was introduced:

10.1109/TIT.1982.1056489

We added the reference.

** Page 7 line 197. Please replace "don't" with "does not".

We replaced “don’t” by “does not”.

** Page 8 line 227. Please replace "gaussian" with "Gaussian".

We replaced “gaussian” by “Gaussian”.

** Page 8 line 251. The symbol "t" in the phrase "when t grows higher" has to be rendered in mathematical font as it denotes the variable t.

We replaced “t” by t.

** Page 9 line 263. Although it is not wrong, I believe it is better to talk about "Monte Carlo simulation" rather than "agent-based model" to indicate the operative implementation of a random walk.

We replaced the “agent-based model” by “Monte Carlo simulation”.

** Page 11 lines 286-287. When describing the implication of the term "small-world" perhaps it would be good to clarify that the clustering coefficient refers to the propensity to form triangles as the use of the term cluster in the rest of the manuscript denotes a different thing and, therefore, might generate confusion in a reader not very familiar with networks. Also, I suggest to cite the work of Watts and Strogatz on the small-world effect (Ref. 21 of the bibliography)

Indeed, we added the clarification of the clustering coefficient as it “refers to the propensity to form triangles, so that nodes that are connected to the same node tend to be also connected between them”

** Appendix S1. I suspect that the phrase "which as the same as" should be "which are the same as". Please, check.

We updated the phrase by “which are the same as”.
